# Sex differences in outcomes of methadone maintenance treatment for opioid addiction: a systematic review protocol

**DOI:** 10.1186/2046-4053-3-45

**Published:** 2014-05-16

**Authors:** Monica Bawor, Brittany B Dennis, Rebecca Anglin, Meir Steiner, Lehana Thabane, Zainab Samaan

**Affiliations:** 1 MiNDS Neuroscience Graduate Program, McMaster University, 1280 Main Street W., Hamilton, ON L8S 4L8, Canada; 2 Population Genomics Program, Chanchlani Research Centre, McMaster University, 1280 Main St. West, Hamilton, ON L8S 4L8, Canada; 3 Health Research Methodology Graduate Program, McMaster University, 1280 Main Street W., Hamilton, ON L8S 4L8, Canada; 4 Department of Clinical Epidemiology and Biostatistics, McMaster University, 1280 Main St. West, Hamilton, ON L8S 4L8, Canada; 5 Department of Psychiatry and Behavioural Neurosciences, McMaster University, 1280 Main St. West, Hamilton, ON L8S 4L8, Canada; 6 Department of Medicine, McMaster University, 1280 Main Street W., Hamilton, ON L8S 4L8, Canada; 7 Women’s Health Concerns Clinic, St. Joseph’s Healthcare Hamilton, 50 Charlton Avenue E., Hamilton, ON L8N 4A6, Canada; 8 Department of Obstetrics and Gynecology, McMaster University, 1280 Main Street W., Hamilton, ON L8S 4L8, Canada; 9 Biostatistics Unit, Centre for Evaluation of Medicine, 25 Main Street W. Suite 2000, Hamilton, ON L8P 1H1, Canada

**Keywords:** Opioid addiction/dependence, Methadone maintenance treatment, Sex differences, Systematic review, Protocol

## Abstract

**Background:**

Use of methadone for the treatment of opioid addiction is an effective harm-reduction approach, although variability in treatment outcomes among individuals has been reported. Men and women with opioid addiction have been known to differ in factors such as opioid use patterns and characteristics at treatment entry; however, little has been reported about differences in methadone treatment outcomes between men and women. Therefore, we present a protocol for a systematic review which aims to provide a summary of existing literature on sex differences in outcomes of methadone treatment for opioid addiction.

**Methods/Design:**

Electronic search of PubMed/MEDLINE, EMBASE, PsycINFO, and CINAHL databases will be conducted using *a priori* defined search strategy. Two authors (MB and BBD) will independently screen potential articles for eligibility using pre-determined inclusion and exclusion criteria and extract key information using a data extraction form designed for this study. Discrepancies will be resolved using a third party (ZS). The primary outcome will be sex differences in response to treatment defined as abstinence from illicit opioid use. We will also assess sex differences in treatment outcomes including treatment retention, remission status post-treatment, polysubstance abuse, methadone dose, drug-related adverse events, health status, psychological status, mortality, criminal activity, high risk sexual behavior, social support/relations, and employment. A meta-analysis will be conducted if possible; risk of bias and overall quality of evidence will be assessed to determine confidence in the estimates.

**Discussion:**

We anticipate that this review will highlight how men and women differ in methadone treatment outcomes and allow us to generate conclusions that can be applied to treatment in a clinical setting.

**Systematic review registration:**

PROSPERO CRD42013006549

## Background

The use of illicit opioids continues to pose a problem both at the individual and societal levels, even more so with the exponentially increasing rates of prescription opioid use in North America [1-3], increasing the risk of development of opioid addiction. Infection [4], medical and psychiatric comorbidity [5], polysubstance use [5], and criminal behavior [6] are among a few of the risks associated with opioid addiction, in addition to a rise in opioid-related deaths [2].

Methadone maintenance treatment (MMT) is the most widely used harm-reduction approach to treating opioid addiction [7]. Methadone is a synthetic analgesic with the ability to inhibit the euphoric effects of opioids and provide relief of withdrawal symptoms due to its longer duration of action [8]. MMT began to receive attention shortly after its development in the early 1940s, which led to the opening of methadone clinics across the world, and later in North America [9]. Since then, the number of patients entering treatment has grown about fivefold [10]. It is estimated that there are >30,000 registered methadone patients in Ontario, Canada, alone [11], which represents approximately 25% of Ontario’s illicit opioid user population [10]. Although progress has been made with MMT, it is evident that it is still not widely used in opioid addiction populations on the larger scale.

Despite the documented effectiveness of methadone as a substitute opioid therapy, methadone has also been reported to produce a large inter-individual variability in response [12], adding an additional layer of complexity to treatment strategies. Traditionally, the population of individuals suffering from opioid addiction has been primarily men, with most studies at the time focusing on opioid-dependent men [13,14]. In the most recent 30 years, there has been an increase in the number of women with opioid addiction [15], which calls for a re-examination of literature on sex differences in opioid addiction in general, and response to MMT specifically.

Sex differences in opioid addiction [10,16-18] and methadone treatment [16,19-22] have been reported; significant sex differences in age, ethnicity, marital status, education, and employment [23], as well as patterns of drug use [21], treatment entry [24], and social support [25] have been identified. Women are typically younger, married, unemployed, and have an earlier onset age of heroin use [23]. Men often use opioids for recreational purposes [16] and have a slower disease progression than women [24]. Additionally, men report earlier treatment entry, more frequent utilization of substance abuse treatment, and fewer psychological and medical problems at treatment admission compared to women [17]. It is becoming clear that treatment needs for men and women are not the same, which points to a demand for separate treatment strategies. The available studies on opioid addiction in the literature are often limited to men [26] or specific ethnic groups, focus on clinical profiles prior to or at treatment entry [16,19-22], or investigate methadone dose as a single outcome of treatment in association with other factors [27-30]. Sex differences have also been examined in opioid addiction patients treated with methadone in association with factors including prescription opioid use [31], drug use patterns [20], drug treatment utilization [32], psychiatric comorbidity [5,33], smoking outcomes [34], and quality of life [35]; however, little has been reported about differences in methadone treatment outcomes between men and women. Few studies have investigated methadone treatment retention, response, remission, adverse events, health status, social relations, criminal activity, and mortality with a specific focus on sex difference, providing inconsistent results and leaving a large gap in the literature with regards to sex differences in response to MMT.

It is also evident that men and women vary in multiple aspects of addiction characteristics and should therefore be provided sex-specific treatment. Implementation of separate treatment approaches for men and women may prove to be a more efficient way to manage this disorder and eventually improve patient-related health outcomes. This review aims to determine whether or not men and women differ in methadone treatment outcomes.

### Objectives

The objective of this review is to summarize the current status of literature regarding sex differences in methadone treatment outcomes by systematically reporting the available research to date. Specifically, we aim to:

1. Assess how men and women differ in methadone outcomes related to drug-use behavior, health status, and sociobehavioral functioning.

2. When suitable, combine the statistical outcomes in a summary estimate through meta-analytical approaches.

3. Critically appraise the literature and determine areas that require further investigation.

## Methods/Design

### Inclusion and exclusion criteria

This systematic review will include completed randomized controlled trials (RCTs) and observational studies of methadone treatment outcomes in men and women. Included studies will focus primarily on sex differences, as opposed to studies on separate populations of men or women. Included studies must also have been conducted in the context of methadone treatment for opioid addiction. Studies including patients that are undergoing a substitute opioid therapy other than methadone (that is, buprenorphine/naloxone, naltrexone) or using methadone for the purpose of detoxification (not maintenance) will be excluded. Studies investigating patient subpopulations such as pregnant women or incarcerated individuals will be excluded as they are too specific to represent the overall population of opioid-dependent individuals and may not allow for the application and generalizability of our findings to community samples. Sex differences in these populations may also be influenced by their environment, leading to a high potential for confounding and bias in the outcomes studied. Patients that are using methadone for the treatment of a condition other than opioid addiction (that is, chronic pain) will also be excluded. Participants shall include both men and women who are receiving methadone treatment for a diagnosis of opioid dependence. No other limitations will be applied (including age or ethnicity) as our intent is to retrieve all articles on sex differences in methadone treatment without restrictions based on population characteristics. The primary outcome of this review will be the presence of sex differences in methadone treatment response, defined as abstinence from illicit opioid use and measured through self-report and/or urinalysis. Sex differences in treatment outcomes will also be assessed with respect to three life domains: drug use-related behavior, health status, and sociobehavioral functioning. These outcomes include treatment retention/duration, remission status post-treatment, polysubstance abuse, methadone dose, drug-related adverse events, health status, psychological status, mortality, criminal activity, high risk sexual behavior, social support/relations, and employment. A complete list of how these outcomes are described, defined, and measured in the literature is available in Table 1.

**Table 1:** Definition of methadone treatment outcomes for assessment

**Outcome**	**Definition**	**Measurement of variable**	**Statistics**	**Studies**
**Drug use-related behavior**				
Response to treatment	Abstaining from illicit opioid use throughout treatment duration	Urine screening	Percentage	[36-39]
		Self reported opioid use (daily or weekly) over specified time period	Mixed model ANOVA Cochran-Mantel-Haenszel statistic	
Treatment retention or duration	Proportion of participants completing treatment; days in treatment from first to last day of therapy	Number of days patient remains in treatment	Cox proportional hazards model	[36-38]
		Proportion of patients retained in treatment for pre-specified duration of study	Kaplan-Meier survival curve	
Remission status post-treatment	Abstinence from use of illicit opioids at follow-up	Urine screening	t-test	[37,39-41]
		Self-reported opioid use (any) after treatment	2x2 factorial ANOVA	
Polysubstance use	Use of at least two (non-opioid) substances throughout the course of treatment	Self-reported use of substances daily or weekly or in last 30 days	Percentage	[37,38]
			Fischer’s Exact Test	
		Net reduction in proportion of drug abuse after specific duration		
**Health and methadone-related outcomes**				
Methadone dose	Average daily methadone dose	Milligrams/day	Difference in means (SD)	[38]
		Mean methadone dose after specific duration in treatment		
Drug-related adverse events	Reaction to treatment drug	Interview/physical examination	Percentage	[17]
		Number of hospitalizations	t-test	
Health status	Change in health status during course of therapy	Interview/physical examination	ANOVA	[17,40]
		Number of hospitalizations		
Psychological status	Comorbidity of psychiatric disorders	Self-reported psychiatric problems	Percentage	[17,42-44]
		Number of reported symptoms	Relative risk	
		Validated psychiatric assessments	ANOVA	
			Chi-square	
Mortality	Treatment-related death or illicit drug use mortality	Mortality causes	Standardized mortality ratio (SMR) Kaplan-Meier survival curve	[45]
		Number of deaths		
		Annual death rate per year of age		
**Sociobehavioral functioning**				
Criminal behavior	Involvement in illegal activities, arrests, or incarcerations throughout treatment or at follow-up	Interview/self-report	Percentage	[17,37,39]
		Current legal status	t-test	
			ANOVA	
High-risk sexual behavior	Involvement in behaviors that put the patient at high risk for HIV and other infections	Use of injection methods (30 days prior)	Weighted least-squares estimation procedure	[46,47]
		Number of sex partners	Repeated measures ANOVA	
		Incidence of unprotected sex		
Social relations/support	Patient’s relationship status and conception of his/her relationship with others	Self-report	ANOVA	[17]
		Number of close friends/family		
		Marital and family status		
		Ratings of interactions		
Employment	Status of employment and evidence of financial income	Change in self-reported employment status during treatment	Percentage	[17,37,39,40]
			Difference in means (SD)	
		Employment status after treatment		
ANOVA, analysis of variance.				

### Search strategy

We shall identify all studies relevant to this review with no language or time restraints. We will search the PubMed/MEDLINE, EMBASE, PsycINFO, and CINAHL databases for relevant articles. Relevant search terms and their medical subject heading (MeSH) equivalents will be used in varying combinations; refer to Table 2 for the complete search strategy. In order to maximize the number of relevant articles retrieved, treatment outcomes will not be included in the search. We will use a wide search to include titles, abstracts, and keyword fields to avoid missing important articles whose title may not reflect the content of the article. Articles will be excluded by limiting the search to humans. We will also manually review reference lists of included studies for studies that may have been missed in the initial search. Grey literature will not be reviewed as we are looking for complete published data only.

**Table 2:** Search strategy for retrieval of relevant articles from multiple databases

**Database**	**Search strategy**
MEDLINE n = 401	1. Opioid-related disorders/dt, rh, th [Drug Therapy, Rehabilitation, Therapy]
	2. Opiate substitution treatment/
	3. Methadone/
	4. Sex Characteristics/
	5. sex differences.m_titl.
	6. gender differences.m_titl.
	7. sex.m_titl.
	8. male.m_titl.
	9. female.m_titl.
	10. men.m_titl.
	11. women.m_titl.
	12. 1 or 2 or 3
	13. 4 or 5 or 6 or 7 or 8 or 9 or 10 or 11
	14. 12 and 13
	15. limit 14 to humans
EMBASE n = 180	1. Opioid-related disorders/dt, rh, th [Drug Therapy, Rehabilitation, Therapy]
	2. Opiate substitution treatment/
	3. Methadone/
	4. Sex Characteristics/
	5. sex differences.m_titl.
	6. gender differences.m_titl.
	7. sex.m_titl.
	8. male.m_titl.
	9. female.m_titl.
	10. men.m_titl.
	11. women.m_titl.
	12. 1 or 2 or 3
	13. 4 or 5 or 6 or 7 or 8 or 9 or 10 or 11
	14. 12 and 13
	15. limit 14 to humans
PsycINFO n = 241	1. exp Methadone Maintenance/ or exp Methadone/
	2. exp Human Sex Differences/
	3. sex.m_titl.
	4. male.m_titl.
	5. female.m_titl.
	6. men.m_titl.
	7. women.m_titl.
	8. 2 or 3 or 4 or 5 or 6 or 7
	9. 1 and 8
	10. limit 9 to humans
CINAHL n = 23	1. Opioid abuse (TX All Text)
	2. Methadone (TX All Text)
	3. Methadone treatment programs (MJ Word in Major Subject Heading)
	4. Gender differences (TX All Text)
	5. Sex differences
	6. 1 or 2 or 3 and 4 or 5
	7. limit 6 to human

### Data screening

Two independent raters (MB and BBD) will screen all citations and abstracts retrieved using the search strategy and identify all eligible articles. Articles that meet the pre-determined criteria will be included for full-text review. Disagreements at any phase of the review process will be resolved by discussion or, in the case where a consensus is not reached, a third independent rater (ZS) will determine eligibility. Ineligible studies will be excluded from the review and reasons for exclusion will be recorded. Inter-rater agreement will be calculated using the Kappa statistic [48] for each phase of screening. Authors will be contacted directly if further data clarification is needed.

### Data extraction

The two authors (MB and BBD) will independently extract data from the studies using a pre-established pilot-tested data extraction form (see Additional file 1). Information obtained will include the author and year of publication, city and country of publication, title of article, journal name, study design, and description of sample population, including total number of men and women study participants, mean age (total and men versus women), and ethnicity. Primary and secondary outcomes, outcome measures, statistical analyses, results, and conclusions will also be recorded. In the case of missing or incomplete data, authors will be contacted for further details. Data will be combined to produce a summary estimate in a meta-analysis if the extracted data allows it.

### Assessment of quality

Two authors (MB and BBD) will independently assess the risk of bias of included studies using the Newcastle-Ottawa Scale (NOS) [49] for observational studies and the Cochrane Collaboration’s tool [50] for assessing risk of bias in RCTs. For observational studies, two authors (MB and BBD) will independently assess the risk of bias of each included study using an adapted version of a modified NOS, specific to the context of this review. This will include seven questions spread across four domains of evaluation; methods for selecting study participants (selection bias), methods to control for confounding (performance bias), statistical methods (detection bias), and methods for measuring exposure and outcome variables (information bias). Risk of bias is measured on a scale of 0 (high risk of bias) to 3 (low risk of bias) and a specific description with examples of both high and low bias is provided. Items regarding selection of participants (representativeness of sample) and ascertainment of outcome (objective versus subjective measures) were retained, while other items relating to the comparability of groups and adequate follow-up for cohort and case-control studies were removed as these were not directly applicable to our topic of interest. We also introduced categories that emphasize statistical methods, confounding effects, and reporting of data to ensure that bias in methodology is assessed. These scales will be used to measure the risk of bias on a per study basis or categorized by domain to develop a general conclusion about the sources of bias in the studies included in this review (see Additional file 2). Cochrane’s tool for assessing risk of bias in RCTs includes seven domains; random sequence generation, allocation concealment, blinding of participants and personnel, blinding of outcome assessment, incomplete outcome data, selective reporting, and other bias. Each of these domains will be evaluated according to high or low risk of bias and will also be assessed on a per study or per domain basis. If a meta-analysis is possible, we will use the Grading of Recommendations, Assessment, Development, and Evaluation framework to rate the quality of evidence through investigation of risk of bias, imprecision (random error), inconsistency, indirectness, and publication bias. We will then summarize the evidence for individual outcomes in summary of findings tables, which will allow for assessment of our confidence in the estimates.

### Statistical analyses and heterogeneity

The results of this systematic review will be reported in a narrative and informative manner; we will discuss issues of study design and statistical analysis methods of the included studies to determine which studies are most informative and reliable. Where possible, we will assess the studies in a combined statistical manner using meta-analysis. The Kappa statistic will be used to measure level of agreement between independent raters. For dichotomous outcomes, we will compute pooled odds ratios using the Mantel-Haenszel random effects model, in which the model is able to estimate between study variation through an evaluation of each study’s final results and a Mantel-Haenszel fixed effect meta-analysis result.

For the summary estimates, we will employ a random effects model, which assumes variation between studies and their respective effect sizes. The nature of observational studies in this population is highly variable, therefore heterogeneity will be accounted for and will allow us to develop aggregate estimates. We will assess the participants, methods, and results of included studies for heterogeneity, which will allow us to determine whether results can be compared across studies. Possible sources of heterogeneity include age groups, study design, methodology, and definition of outcome. In case of heterogeneity, subgroup analyses according to these different categories will be performed. Included studies will be presented in the form of a forest plot. We will use Review Manager 5.1 software (The Cochrane Collaboration, London, UK) for all statistical analysis and results will be presented using 95% confidence intervals.

### Presenting and reporting of results

We will report the systematic review according to the Preferred Reporting Items for Systematic reviews and Meta-Analyses guidelines [51]. A flow diagram will be used to summarize the selection process of studies at each phase and summary tables will be used to report study characteristics and presence of sex differences per methadone outcome. Publication bias will also be examined and assessed using Egger’s plot.

## Discussion

Using evidence from this systematic review, we expect to draw conclusions regarding the presence of sex differences in outcomes of MMT for opioid addiction. This review will not only provide us with summary evidence for which we can objectively make inferences about the current status of literature, it will also allow us to critically evaluate the methodological quality and risk of bias present in the available evidence. The literature on methadone treatment focuses primarily on men and little is known about women or how the sexes compare. We anticipate that this review will highlight how men and women differ in methadone treatment outcomes and allow us to generate conclusions that can be applied to treatment in a clinical setting. We will encourage healthcare professionals to make use of this information and approach men and women dealing with opioid addiction using different treatment strategies, catered to each sex specifically. We are hopeful that this review will ultimately establish the need for further examination into sex differences in methadone treatment in an effort to improve treatment prognosis for individuals dealing with this complex disorder.

## Abbreviations

MMT: methadone maintenance treatment; NOS: Newcastle-Ottawa Scale; RCT: randomized controlled trial.

## Competing interests

The authors declare that they have no competing interests.

## Authors’ contributions

MB: conception and design, manuscript writing, critical revision, development of data extraction forms and quality assessment tools, and final approval of manuscript. BBD: interpretation and methodology, help with development of quality assessment tool, critical revision, and final approval of manuscript. RA: consultations for search strategy and quality assessment, critical revision, and final approval of manuscript. MS: interpretation of literature, critical revision, and final approval of manuscript. LT: methodology, critical revision, and final approval of manuscript. ZS: conception and design, critical revision, methodology, and final approval of manuscript. All authors read and approved the final manuscript.

## Supplementary Material

Additional file 1 **Data extraction form for included studies.** This form contains the information which we intend to extract from included studies during the data extraction process. It includes general study information, methods and description of sample, outcomes, and results. Click here for file

Additional file 2 **Adapted version of a modified Newcastle-Ottawa Scale (NOS) for single use in specific context.** This form demonstrates the modified version of the NOS categorized by domain of evaluation and supported with examples of levels of bias. Observational studies will be assessed on risk of bias based on this form. Click here for file
