# Supplementary material for: Sex differences in outcomes of methadone maintenance treatment for opioid addiction: a systematic review protocol
Source: Syst Rev. 2014 May 16;3:45. doi: 10.1186/2046-4053-3-45 (PMC4031161; doi:10.1186/2046-4053-3-45)
Supplement: Additional file 1 — Data extraction form for included studies. This form contains the information which we intend to extract from included studies during the data extraction process. It includes general study information, methods and description of sample, outcomes, and results. [file 2046-4053-3-45-S1.pdf]

SYSTEMATIC REVIEW FULL-TEXT EXTRACTION FORM

STUDY INFORMATION

Study ID: _________ **Reviewer Initials:** _________

Last Name of First Author, Initial: _____________________________________ **Year of Publication:** ____________

Title of Article: ____

Journal Name: _____________________________________ **City, Country:** _____________________________________

METHODS

Study Setting: _____________________________________ **Study Design:** ______________________________________

Sample Size: Total __________, Men _________, Women _________

Mean Age (SD): Total ______________, Men ______________, Women ______________

Ethnicity: _____________________________________

RESULTS

MMT-related Outcome (See List of Outcomes in Protocol): _______________________________________

Specific domain of outcome (See List of Domains in Protocol): _____________________________________

Outcome Definition: ____

Outcome Measurement: ____

Outcome Values: Men ______________, Women ______________

Statistical Testing/Methods: _____________________________________

Statistical Results: Coefficient ______________, 95% CI ______________, p-value ______________

Study Limitations: ____

Inclusion [must check all to be included]

- ☐ **The study is looking at MMT patient populations**
- ☐ **Study participants on MMT for the treatment of opioid addiction**
- ☐ **The study participants are human**
- ☐ **The study has been completed**
- ☐ **The study is in English**

Exclusion [exclude study if any of the following is checked]:

- ☐ **Incomplete studies**
- ☐ **Abstract only**
- ☐ **The primary focus of the study is not MMT-related**
- ☐ **The study focuses on a different Substitute Opioid Therapy (i.e. suboxone)**
- ☐ **The study is on animal populations**

Comments: ____
